# Marine Mammals’ NMDA Receptor Structure: Possible Adaptation to High Pressure Environment

**DOI:** 10.3389/fphys.2018.01633

**Published:** 2018-11-22

**Authors:** Alice Bliznyuk, Hava Golan, Yoram Grossman

**Affiliations:** Department of Physiology and Cell Biology, Faculty of Health Sciences and Zlotowski Center for Neuroscience, Ben-Gurion University of the Negev, Beersheba, Israel

**Keywords:** NMDA receptor, high pressure, cetacean, HPNS, GluN2A

## Abstract

Divers that are exposed to high pressure (HP) above 1.1 MPa suffer from High Pressure Neurological Syndrome (HPNS), which is implicated with central nervous system (CNS) malfunction. Marine mammals performing extended and deep breath-hold dives are exposed to almost 20 MPa without apparent HPNS symptoms. *N*-methyl-D-aspartate receptor (NMDAR) has repeatedly been implicated as one of the major factors in CNS hyperexcitability as part of HPNS. Electrophysiological studies in rat brain slices at He HP showed a significant increase in the synaptic NMDAR response, followed by postsynaptic excitability changes. Molecular studies of *Rattus norvegicus* NMDARs have revealed that different subunit combinations of the NMDAR exhibit different, increased or decreased, current responses under He HP conditions. The purpose of the present research was to disclose if the breath-hold deep diving mammals exhibit NMDAR structural modifications related to HP. We used sequence alignment and homology structure modeling in order to compare deep diving marine mammals’ NMDARs to those of terrestrial mammals. We discovered that deep diving mammals have a special tertiary TMD structure of the GluN2A subunit that differs from that of the terrestrial mammals. In addition, the GluN2A subunit has a group of four conserved a.a. substitutions: V68L (*N-*terminal domain, NTD) and V440I (agonist-binding domain, ABD) are cetacean specific, E308D (*N*-terminal domain, NTD) and I816V (transmembrane domain, TMD) were also singularly found in some terrestrial mammals. Since I816V is localized in M4 α-helix region, which is critical for NMDAR activation and desensitization, we hypothesize that the presence of all 4 substitutions rather than a single one, is the combination that may enable HP tolerance. Furthermore, additional special substitutions that were found in the marine mammals’ NTD may affect the Zn^2+^ binding site, suggesting less or no voltage-independent inhibition by this ion. Our molecular studies of NMDARs containing the GluN2A subunit showed that HP removal of the Zn^2+^ voltage-independent inhibition could be the mechanism explaining its current increase at HP. Thus, this mechanism could play a crucial role in the CNS hyperexcitability at HP. Less or no voltage-independent Zn^2+^ inhibition, different conformations of the TMD, and special mutation in the M4 α-helix region of cetaceans’ NMDAR, may give them the advantage they need in order to perform such deep dives without CNS malfunction.

## Introduction

Professional divers may suffer from direct high pressure (HP) effects that present many physiological challenges, especially affecting the lungs, hollow viscera, and nervous system. Animals and humans exposed to ambient pressure above 0.2 MPa may suffer from O_2_ toxicity, which is thought to occur through increased oxidative stress. HP above 0.5 MPa induces N_2_ narcosis (inert-gas narcosis) as well as CO_2_ toxicity. Divers that are exposed to HP above 1.1 MPa suffer from the High Pressure Neurological Syndrome (HPNS) (Halsey, 1982; Tibbles and Edelsberg, 1996; Bennett and Rostain, 2003; Grossman et al., 2010). HPNS is characterized by reversible central nervous system (CNS) hyperexcitability and cognitive and motor deficits. Symptoms of HPNS include tremors, myoclonic jerking (Darbin et al., 2000), somnolence, EGG changes, visual disturbance, nausea, dizziness, and decreased mental performance (Brauer, 1984; Abraini, 1997). HPNS susceptibility and symptoms’ intensity depend on the compression rate and on the absolute ambient pressure. All but the HPNS can be alleviated and even eliminated by controlling partial-pressures of absorbed tissue gasses at normal values while under HP conditions by using a variety of devices.

Marine mammals performing extended and deep breath-hold dives while foraging was described decades ago (Irving, 1939). Although the research on their capability of storing and carrying O_2_ has been expanded, little is known about their CNS adaptation for that. It is still unknown how the marine mammals are capable of performing deep dives to an extreme depth in a very short time without suffering from HPNS symptoms. Sperm whale males can reach the depth of 1860 m ( ∼19 MPa) and perform seven dives to such depths during 9 h of foraging behavior (Teloni et al., 2008). Similar behavior has been reported for northern elephant seals (DeLong and Stewart, 1991). In general, many marine mammals can perform sequences of alternate deep and fast dives followed by somewhat slower ascents and relatively short surface time (see Table 1 for maximal diving depth of different animals). Such dive protocols cannot be performed by humans or other terrestrial mammals without causing death or other irreversible consequences.

**Table 1:** List of the sequences obtained from the Gene/Protein-NCBI data bank.

Organism	Species	Gene ID	Gene symbol	Protein ID	Estimated maximum diving depth (m)	Retrieved on (date)
Human	*Homo sapiens*	2902	grin1	NP_015566.1	NA	2017 October
	(Primates)	2903	grin2a	NP_000824.1		
		2904	grin2b	XP_011518931.1		
Rat	*Rattus norvegicus*	24408	grin1	NP_058706.1	NA	2017 October
		24409	grin2a	NP_036705.3		
	(Rodentia)	24410	grin2b	NP_036706.1		
Goat	*Capra hircus*	102181789	grin1	XP_017911741.1	NA	2018 July
	(Artiodactyla)	102189537	grin2a	XP_017896494.1		
		108636054	grin2b	XP_017903945.1		
Cow	*Bos Taurus*	100336579	grin1	XP_024855439.1	NA	2018 August
	(Artiodactyla)					
		112444298	grin2a	XP_024841004.1		
		537804	grin2b	NP_001179850.1		
Dog	*Canis lupus familiaris* (Carnivora)	494007	grin1	NP_001008717.1	NA	2018 August
		490012	grin2a	XP_005621613.1		
		494009	grin2b	NP_001008719.1		
African elephant	*Loxodonta africana* (Proboscidea)	100663779	grin1	XP_023398181.1	NA	2018 August
		100658424	grin2a	XP_023413048.1		
		100660738	grin2b	XP_023409909.1		
Arabian camel	*Camelus dromedaries* (Artiodactyla)	105106183	grin1	XP_010998322.1	NA	2018 August
		105089056	grin2a	XP_010978357.1		
		105096536	grin2b	XP_010987193.1		
Rabbit	*Oryctolagus cuniculus*	100337889	grin2a	XP_008256030.1	NA	2018 August
		100353266	grin2b	XP_008257825.1		
	(Lagomorpha)					
Sperm whale	*Physeter catodo n*	102987558	grin1	XP_007103722	1860	2017 October
					(Teloni et al., 2008)	
	(Cetaceans)	102993136	grin2a	XP_007128477		
		102992015	grin2b	XP_007125880.1		
Beluga whale	*Delphinapterus leucas*	111176991	grin1	XP_022433790.1	872	2017 October
		111163530	grin2a	XP_022407990.1	(Heide-Jorgensen et al., 1998)	
	(Cetaceans)	111176787	grin2b	XP_022433346.1		
Killer whale	*Orcins orca*	101278622	grin1	XP_004285003.1	260	2017 October
	(Cetaceans)	101270526	grin2a	XP_004270238.1	(Berta et al., 2015)	
		101285554	grin2b	XP_004281176.1		
Minke whale	*Balaenoptera acutorostrata scammoni*	103001022	grin1	XP_007182793.1	150	2017 October
		103006151	grin2a	XP_007188474.1	(Christiansen et al., 2015)	
		102999259	grin2b	XP_007196105.1		
	(Cetaceans)					
Bottlenose dolphin	*Tursiops truncatus*	101331481	grin2a	XP_019784187.1	535	2017 October
		101337480	grin2b	XP_019793998.1	(Berta et al., 2015)	
	(Cetaceans)					
Yangtze River dolphin	*Lipotes vexillifer*	103085162	grin1	XP_007459011.1	Unknown	2017 October
	(Cetaceans)	103091670	grin2a	XP_007462899.1		
		103082207	grin2b	XP_007454696.1		
Yangtze finless porpoise	*Neophocaena asiaeorientalis asiaeorientalis*	112409459	grin2a	XP_024615377.1	Unknown	2018 July
	(Cetaceans)	112391785	grin2b	XP_024589012.1		
						
Hawaiian monk seal	*Neomonachus schauinslandi*	110581308	grin1	XP_021546770.1	300	2018 July
		110587916	grin2a	XP_021553823.1	(Delong et al., 1984)	
	(Pinnipeds)	110580897	grin2b	XP_021546258.1		
Weddell seal	*Leptonychotes weddellii*	102729649	grin1	XP_006737427.1	626	2018 July
		102749455	grin2a	XP_006734583.1	(Castellini et al., 1992)	
						
	(Pinnipeds)	^∗^gene position:	grin2b	unavailable		
		KB715431:718545-1018531				
Pacific walrus	*Odobenus rosmarus divergens*	101368224	grin1	XP_004416702.1	80	2018 July
					(Schreer et al., 2001)	
		101378928	grin2a	XP_004401310.1		
		101365544	grin2b	XP_004411138.1		
	(Pinnipeds)					
Florida Manatee	*Trichechus manatus latirostri*	101349517	grin1	Presently unavailable	16	2018 July
		101353799	grin2a	XP_004373303.1	(Edwards et al., 2016)	
	(Sirenians)	101340402	grin2b	Presently unavailable		
						


*Protein ID, NP indicates protein verification; XP indicates DNA model prediction. ^∗^The GluN2B of Weddell seal was obtained from UCSC genome browser: https://genome.ucsc.edu/cgi-bin/hgc?hgsid=687847867_07mUD2aKrRC1BVbck0azZZ53jFZS&c=KB715431&l=718544&r=1018531&o=718544&t=1018531&g=hub_83043_CESAR_Annotations&i=GRIN2B*

*N*-methyl-D-aspartate receptor (NMDAR) belongs to the family of ionotropic glutamate receptors (iGluRs) that mediate excitatory neuronal transmission within the CNS. Earlier pharmacological studies have repeatedly implicated the NMDAR in CNS hyperexcitability as a major part of HPNS (Fagni et al., 1985, 1987; Zinebi et al., 1988, 1990; Daniels and Grossman, 2003; Mor and Grossman, 2010). In contrast, other iGluRs members such as the α-amino-3-hydroxy-5-methyl-4-isoxazolepropionic acid receptor (AMPAR) contribute a very small part in inducing the CNS hyperexcitation at HP (Pearce et al., 1994), the kainite response was even unaffected by pressure at all (Shelton et al., 1993). Other a.a. ionotropic receptors such as GABA are also insensitive to HP (Roberts et al., 1996) and glycine maximal response is not changed, but IC_50_ was considerably increased at HP (Shelton et al., 1993). Interestingly, the hypothesis of NMDAR regulation being involved in the limitation of physiological tolerance to both low temperature and high hydrostatic pressure in marine crustaceans has been recently suggested (Brown and Thatje, 2018).

**FIGURE 1:**
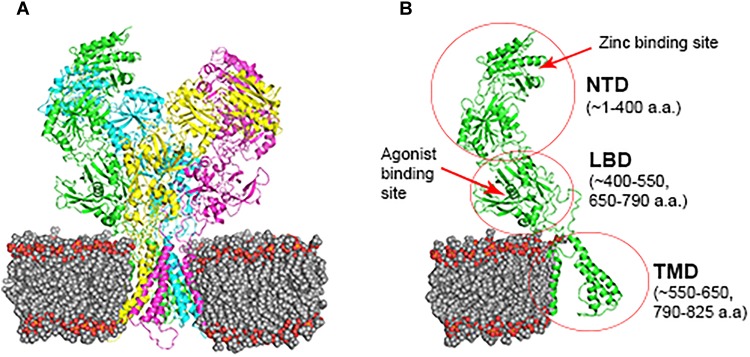
NMDAR subunit structure. All GluN subunits share a modular architecture that is composed of four distinct domains: the N-terminal domain (NTD), the agonist-binding domain (ABD) that binds to glycine or d-serine in GluN1 and GluN3, and glutamate in GluN2, the transmembrane domain (TMD) containing the ion channel, and an intracellular C-terminal domain (CTD; not indicated in the figure). The NTD and CTD are the most divergent regions. **(A)** NMDAR in the membrane, each subunit is indicated by different color using cartoon representation. **(B)** One subunit of the NMDAR in the membrane, important sites are indicated by arrows. Numbers in parenthesis indicate the a.a. span of the domain (red circle).

There are 14 different subunits of NMDARs (Traynelis et al., 2010): the eight GluN1-1a to -4a and GluN1-1b to -4b subunits result from alternative RNA splicing (Dingledine et al., 1999). The four GluN2A to GluN2D subunits are encoded by four different genes, while the two GluN3A and GluN3B subunits are encoded by two genes. Conventional NMDARs are assembled from different combinations of GluN1 and GluN2 subunits in a heterotetrameric “dimer of dimers” structure (Furukawa et al., 2005; Paoletti, 2011). At least one GluN1 subunit must be incorporated into the receptor to enable its transportation to the membrane. Different NMDAR subtypes have specific spatiotemporal distribution and function(s) in the CNS (Watanabe et al., 1992; Akazawa et al., 1994; Laurie and Seeburg, 1994; Monyer et al., 1994; Sheng et al., 1994; Takai et al., 2003; Paoletti, 2011). Studies on recombinant, heterologously expressed NMDARs have revealed how the subunit composition endows each subtype with unique biophysical and pharmacological properties (Paoletti et al., 2013; Sanz-Clemente et al., 2013). NMDARs are highly permeable to Ca^2+^ but also can transmit Na^+^ and K^+^. Ca^2+^ influx through NMDARs plays an important role in learning and long-lasting changes in synaptic efficacy such as long-term potentiation (LTP) and long-term depression (LTD) (Collingridge et al., 2004). Each NMDAR subunit contains large extracellular N-terminal domain (NTD); agonist-binding domain (ABD); and transmembrane domain (TMD) that has three membrane-spanning domains, a re-entry loop that forms the pore-lining region (membrane domain 2), and an intracellular carboxy C-terminal domain (CTD) (Traynelis et al., 2010) (see Figure 1 for NMDAR structure). The GluN2A and GluN2B subunits are the most abundant in adult CNS. GluN2A has a fast kinetics, high open probability, fast deactivation time whereas the GluN2B has the opposite characteristics (Traynelis et al., 2010; Paoletti, 2011). During LTP the amount of the NMDARs containing the GluN2A subunit in the hippocampal CA1 region of adult animals increases and the ratio between GluN2B and GluN2A inverts (O’Brien et al., 1998; Kim and Sheng, 2004). NMDARs containing GluN2A are inhibited by nM concentrations of Zn^2+^, which is termed the high-affinity voltage-independent Zn^2+^ inhibition (Paoletti et al., 1997; Romero-Hernandez et al., 2016). The Zn^2+^ voltage-independent site is positioned in the NTD region and contains four residues: H44, H128, E266, and D282 (Romero-Hernandez et al., 2016).

Electrophysiological studies in rat brain slices at He HP, preceded by the pharmacological studies, showed a significant increase in the synaptic NMDAR response followed by postsynaptic excitability changes (Mor and Grossman, 2006; Mor and Grossman, 2007). Lately, molecular studies from our laboratory (Mor et al., 2012; Bliznyuk et al., 2015, 2016) have revealed that different subunit combinations of the NMDAR exhibit different current responses, an increase or a decrease, under He HP conditions. NMDAR containing GluN2A subunit showed a 25–60% current increase at HP (dependent on GluN1 subunit type), whereas the receptors containing GluN2B subunit did not change. Taking together the data on NMDARs and on other CNS receptors, strongly indicates that NMDAR containing the GluN2A subunit is the only member of the iGluRs that contributes to CNS hyperexcitation at HP. Lately it has been shown by our laboratory (Bliznyuk et al., 2017) that HP removes the voltage-independent Zn^2+^ inhibition of the receptor.

The ability of the diving mammals to perform an extreme deep and fast dive without any consequences strongly implies that deep diving mammals possess several “adaptive” mechanisms (especially neurophysiological ones, such as NMDAR modifications) that increase their pressure tolerance. The purpose of this research was to disclose whether the breath-hold deep diving mammals exhibit NMDAR structural changes that may be beneficial for coping with HP environment. We used sequence alignment and homology structure modeling in order to compare deep diving marine mammals’ NMDARs to those of terrestrial mammals.

## Materials and Methods

### Sequence Alignment

Presently available data on diving marine mammals was obtained from the Gene-NCBI data bank and UCSC Genome Browser (Table 1). Protein sequences of the marine mammals were aligned to terrestrial mammals (human, rat) while goat, cow, dog, African elephant, Arabian camel, and rabbit served as additional controls. The choice of human as a terrestrial mammal is obvious, in order to find out whether genetic management of HP effects is feasible. We chose rat as an additional mammal because of its high gene matching to the human genome, and also because the electro-physiological research carried out in our lab was done mainly on rat NMDAR mRNAs (Mor et al., 2008; Bliznyuk et al., 2015, 2016). Alignment was performed by Jalview 2.10.3 program (Waterhouse et al., 2009) using a web server running T-Coffee with defaults. The full alignments can be found in Supporting Supplementary Figures S1–S3.

The numbering of a.a. presented in the study matches the number each a.a. received after the alignment.

### Homology Structure Modeling

The Phyre2 web portal for protein modeling, prediction, and analysis was used in order to predict the tertiary structure of the proteins (Kelley et al., 2015). The 3D predictions that were chosen had a level of confidence of ∼90–100% (i.e., the probability that the sequence and the template are homologous) and % i.d. (the percentage identity between the sequence and the template) above 40% (indicates high accuracy models).

PyMOL software (Schrödinger, 2015) was used to:

• Perform 3D structure alignment, not including the CTDs because of unknown 3D structure.
• Calculate Root Mean Square Deviation (RMSD), the square root of the mean of the square of the distances between the matched atoms, of the 3D structure alignment. Since the GluN2A alignment of human to rat yielded RMSD = 0.268, we consider RMSD < 0.3 to be very good alignment, 0.3–0.8 as moderate, 0.8–1.0 poor, and >1.0 very poor alignment. RMSD is a useful tool to numerically evaluate the visual differences of the 3D structure of 2 proteins.
• Create images of molecular structures.

## Results

Protein sequences of diving marine mammals were aligned to those of chosen terrestrial mammals (rat and human). Other terrestrial mammals such as goat, cow, dog, African elephant, Arabian camel, and rabbit served as additional controls for specific diving mammals. House mouse and chimpanzee were also examined and presented high sequence similarity to the terrestrial mammals and therefore omitted from the study. We also checked chicken and Emperor penguin (known as a deep diver), and even frog and Whale shark, whose sequences were dissimilar to mammals and therefore excluded from the study.

### GluN1 Family Conservation

**FIGURE 2:**
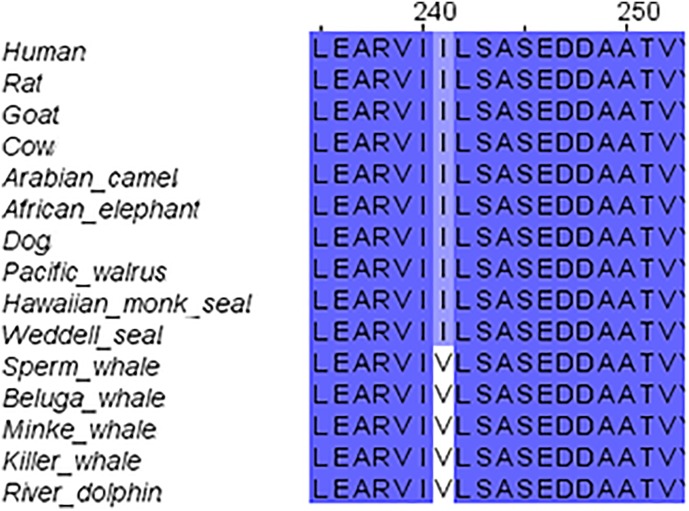
Sequence alignment of GluN1 subunit in marine and terrestrial mammals. Valine (V) 241 is conserved across all tested cetaceans; it replaced Isoleucine (I) in all other mammals.

We started our systematic analysis of all NMDARs subunits by alignment of the GluN1 family (GluN1-1) of marine mammals in comparison to those of the terrestrial mammals. The GluN1-1 was found highly conserved (not including the CTD because of the alternative splicing of this subunit domain) except for one a.a. I241 that was substituted for V in the cetaceans (Figure 2). It is necessary to note that the Sperm whale sequence is not complete and contains a big gap in the TMD region. Sequences of the Bottlenose dolphin and Finless porpoise are incomplete and contain multiple gaps (only 177 a.a. of the ∼938 a.a. sequence) and therefore were excluded from the alignment. Florida manatee and rabbit GluN1 subunit is presently unavailable in NCBI. However, structure modeling of the available subunits showed that the small differences and the conserved mutation I241V in cetaceans do not influence the tertiary structure of the subunit (not shown).

### GluN2 Family

#### GluN2B Conservation

**FIGURE 3:**
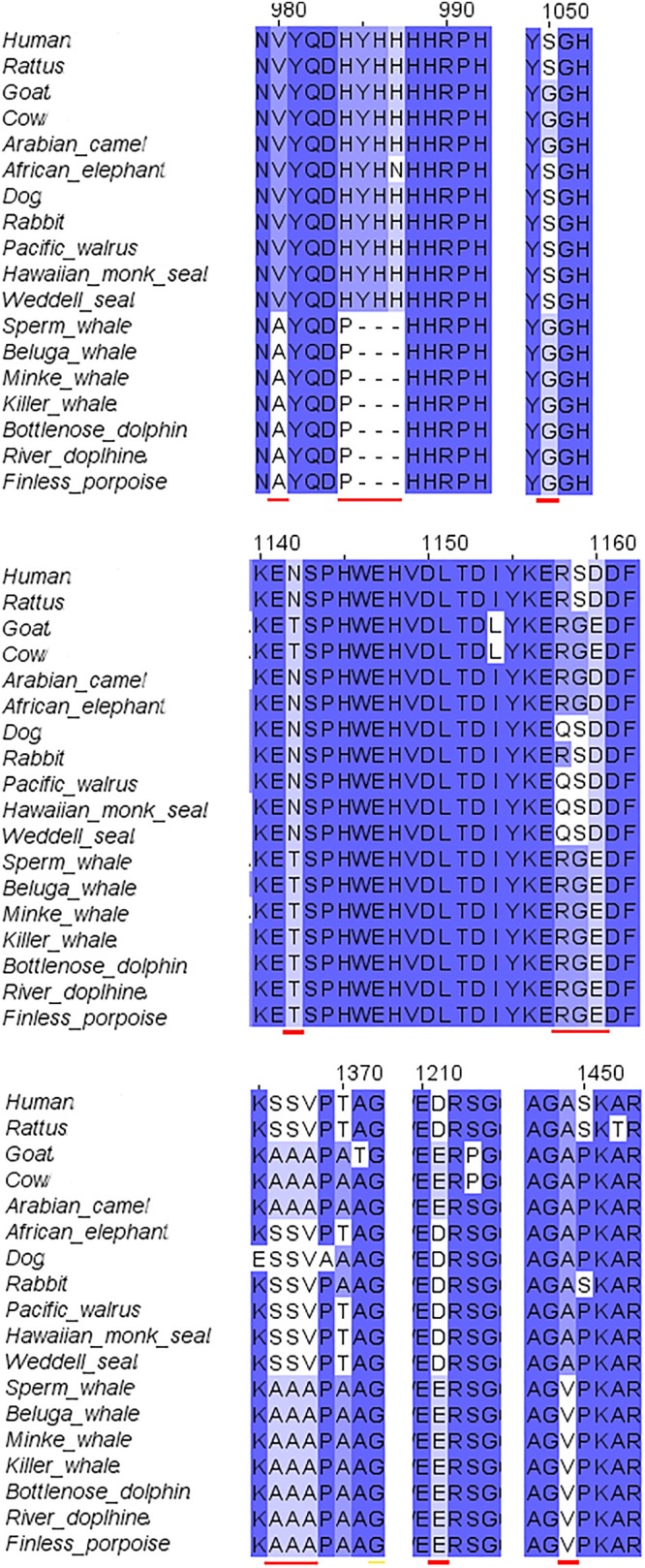
Sequence alignment of GluN2B subunit in marine and terrestrial mammals. A lot of the a.a. substitutions and deletions (indicated with red lines) in the cetaceans were observed in the *C*-terminal domain, only some of them were also replaced in goat.

The second step of our study was the alignment of the GluN2B subunit. It revealed that there are no special substitutions or deletions in NTD, ABD, or TMD of the receptor. However, cetacean family members showed a special replacement in the CTD (V980A, H983P, deletion of YHH in 985-988 and A1449V). Also special replacements for cetacean and their close relatives goat, cow and camel were observed (S1050G, N1142T, D1160E, S1367A, V1368A, D1211E) (see Figure 3 for details). Florida manatee GluN2B subunit is presently unavailable in NCBI. The alignment of the available subunits indicated that the tertiary structure of the subunit is fully conserved (not shown).

#### GluN2A Differences

**FIGURE 4:**
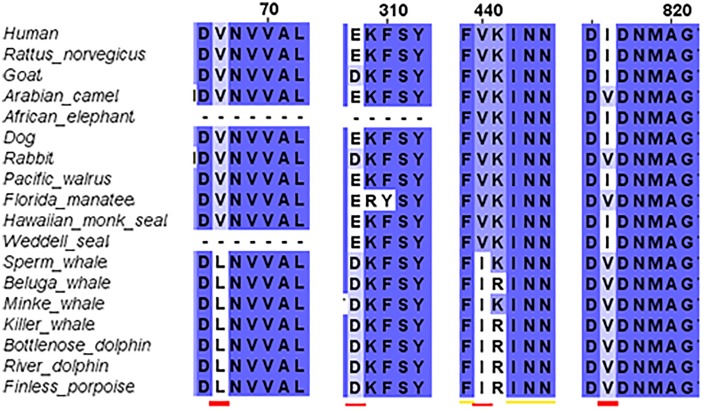
Sequence alignment of GluN2A subunit in marine and terrestrial mammals. Special substitutions of the a.a. in the cetaceans were observed: V67L (in NTD), E308D (in NTD) appears also in goat, V440I (in ABD), and I816V (in TMD, α-helix M4) appears also in Florida manatee.

The third step of the full protein alignment dealt with GluN2A. It revealed four specific substitutions of a.a. in cetaceans (see Figure 4): V67L (in NTD), E308D (in NTD), V440I (in ABD), and I816V (in TMD, α-helix M4). E308D was also substituted in Goat and rabbit, I816V in Florida manatee, rabbit and camel. Sequence of the cow is incomplete (only 761 a.a. of the ∼1464 a.a. sequence) and therefore, was excluded from the analysis (nonetheless can be seen in Supplementary Figure S3).

##### Tertiary prediction differences

**FIGURE 5:**
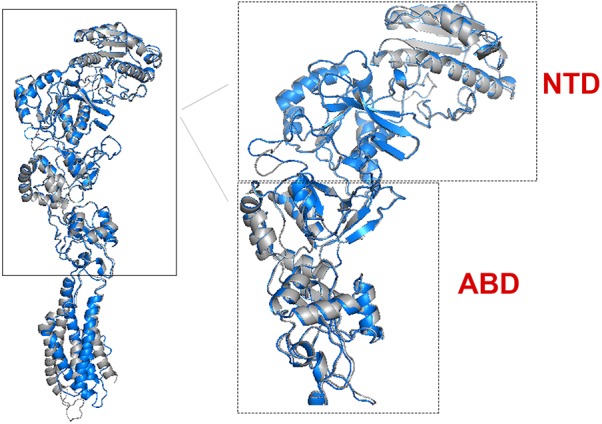
Predicted tertiary structure alignment of GluN2A subunit in Sperm whale (blue) and rat (gray). NTD and ABD demonstrated high alignment conservation levels compared to rat.

The tertiary structure prediction, using the Phyre2 web portal of the subunit, showed that there is a very good conservation of the NTD and ABD in all marine mammals in comparison with rat (*Rattus norvegicus*) (Figure 5). The average RMSD (see method for explanation) for NTD and ABD calculated together was 0.323; for comparison, RMSD for human was 0.268. Unfortunately Weddell seal (known as a good diver, 626 m) lacks half of the NTD sequence (121 a.a.) for unknown reasons (either technical or biological), and therefore we could not evaluate its NTD sequence prediction.

**FIGURE 6:**
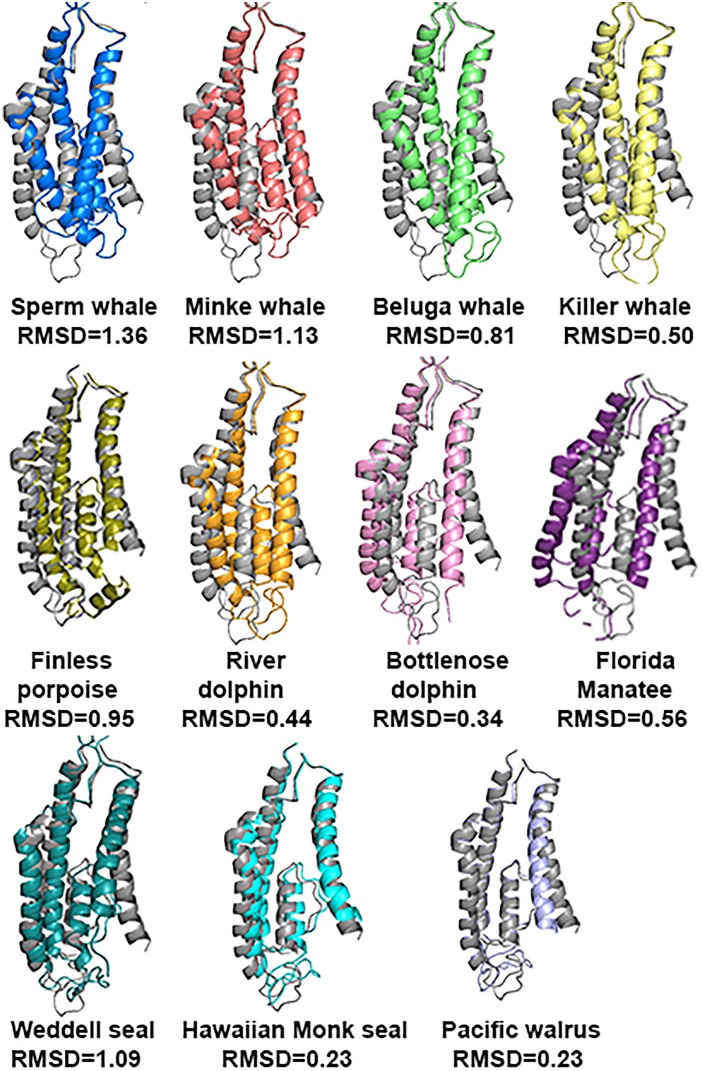
Predicted tertiary structure alignment of TMD of GluN2A subunit in the marine mammals and rat (gray). Sperm whale (the deepest diver 1860 m) and Weddell seal (626 m) presenting the lowest degree of conservation.

However, the TMD that contains the pore of the receptor has a different 3D structure (Figure 6) and it does not align with a similar RMSD value with the rat TMD. The most striking differences of the TMD structure were observed in Sperm whale (RMSD = 1.359) and Weddell seal (RMSD = 1.09), which are deep divers in their groups (1865 and 626 m, respectively). To our surprise, Florida manatee showed a considerable difference in TMD (RMSD = 0.56) although it is considered a shallow diver. It is important to note that despite the single substitution (E308D) in the goat, the TMD structure was conserved (not shown) as indicated by low RMSD (0.324).

##### GluN2A Zn^2+^ conformation differences of the site-prediction

**FIGURE 7:**
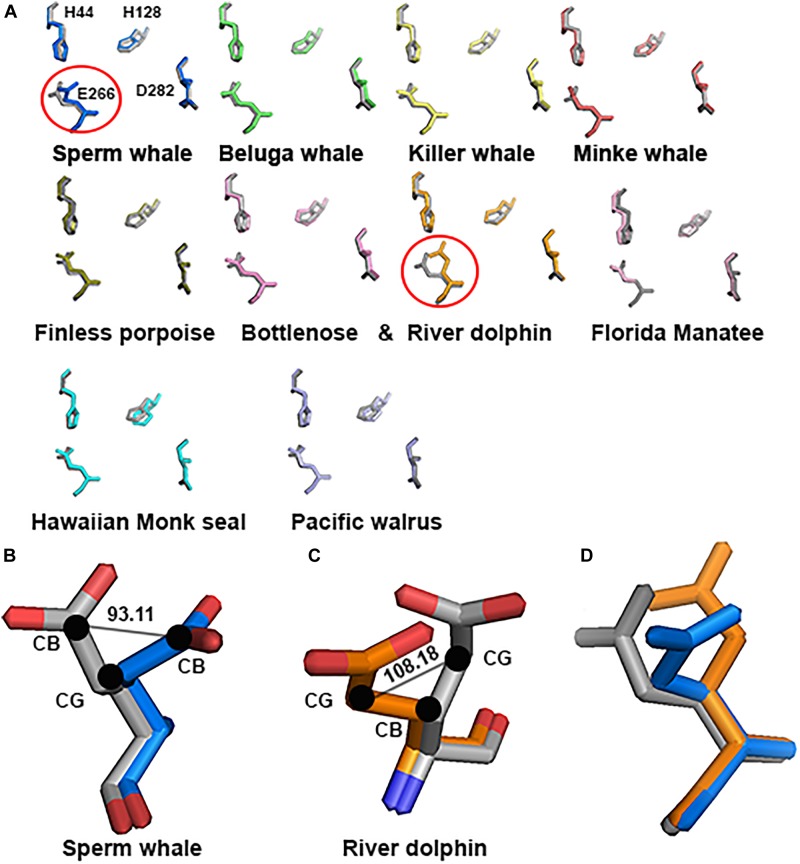
Zn^2+^ site prediction of the marine mammals’ alignment to rat (gray). **(A)** The four residues of the Zn^2+^ site responsible for binding the Zn^2+^ ion are represented by sticks. Note the major change in E266 residue (indicated by circle) in Sperm whale and River dolphin. **(B,C)** Zoom in to the E266 of the Sperm whale and River dolphin alignment to rat (gray), represented by sticks and colored by element. Angle of the rotation represented in degrees, black dots indicate the carbon and its index next to it. **(D)** Alignment of the E266 residue of rat (gray), Sperm whale (marine) and River dolphin (orange) represented by sticks in the same plane as in **A**. Note that the rotation in Sperm whale and River dolphin is in different carbons and on a different plane.

As mentioned in the Introduction, NMDARs containing GluN2A are inhibited by nM concentrations of Zn^2+^, which is termed the high-affinity voltage-independent Zn^2+^ inhibition. We have recently shown (Bliznyuk et al., 2017) that HP removes this type of Zn^2+^ inhibition. Based on this conclusion, we decided to check the alignment of this specific site. Sequence alignment revealed that the site sequence (four residues H44, H128, E266, and D282 that are responsible for Zn^2+^ binding) is conserved. However, only one E266 has a different conformation in Sperm whale and River dolphin (Figure 7A). Sperm whale has a 93.11° rotation on the CG atom (Figure 7B), River dolphin has a rotation on the CB atom of 108.18° (Figure 7C). The rotation in the Sperm whale is on a different plane than that of the River dolphin rotation (see Figure 7D). Unfortunately, Weddell seal lacks two a.a. (H44 and H128) of this site due to the above mentioned deletion in NTD and therefore was not analyzed.

#### Substitutions T156I, S224T, V67L, E308D, V440I, and I816V

In order to investigate whether the 4 specific substitutions discovered in cetaceans (V67L, E308D, V440I, I816V) and the special TMD conformation are responsible for their fitness to HP environment, we changed those residues in the sequence of the rat and predicted its tertiary structure.

**FIGURE 8:**
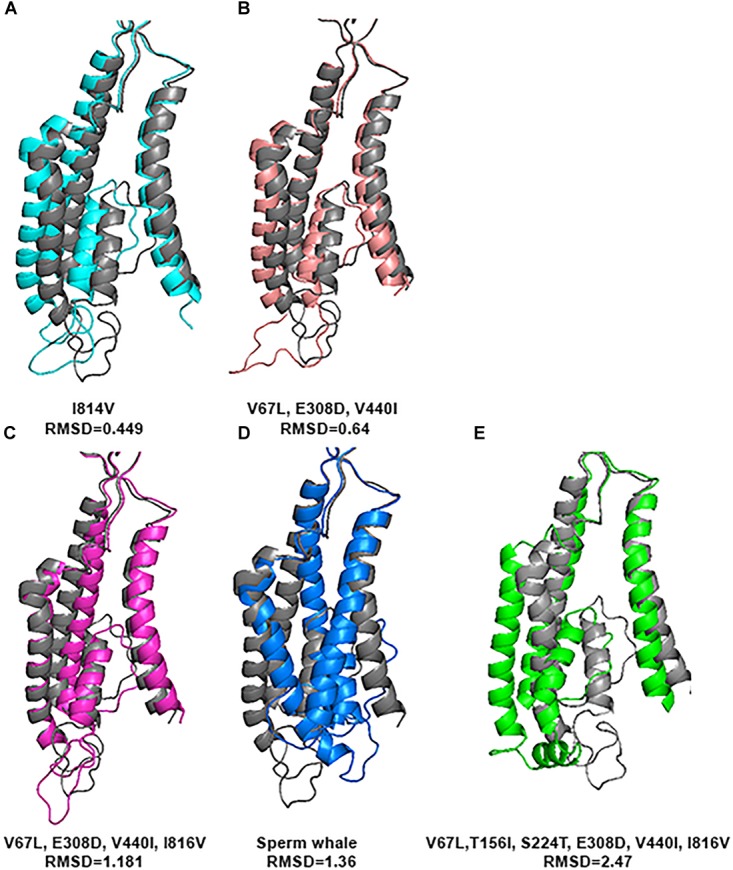
Predicted tertiary structure of TMD alignment of the substitution of GluN2A subunit and GluN2A subunit of the rat (gray). **(A)** I814V. **(B)**V67L, E308D, V440I. **(C)** V67L, E308D, V440I, I816V. **(D)** Sperm whale. **(E)** V67L, T156I, S224T, E308D, V440I, I816V. Note that the sperm whale TMD conformation **(D)** is more distorted compared to the rat possessing the same four substitutions **(C)**. Rat TMDs possessing only one **(A)** and three **(B)** substitutions (indicated below each alignment) are even less distorted compared to the native rat.

**FIGURE 9:**
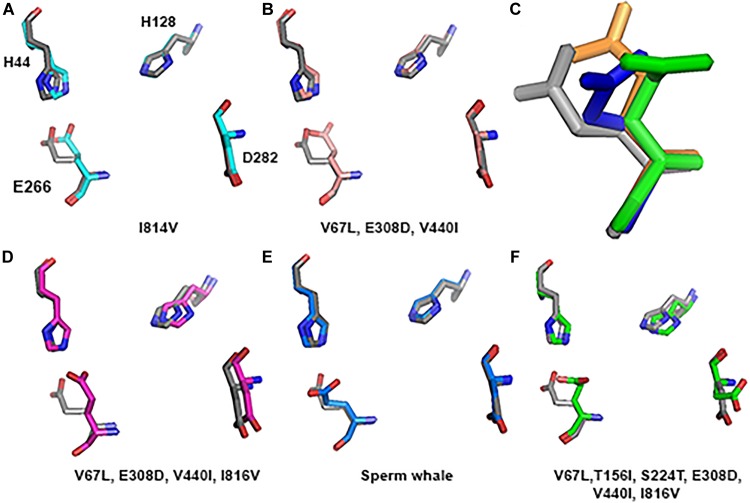
Zn^2+^ site prediction of the substituted subunit GluN2A alignment to rat (grey**A**–**B** and **D**–**F**). The four residues of the Zn^2+^ site that are responsible for binding the ion are represented by sticks. **(C)** Zoom in to the E266 of the Sperm whale (blue), six substitutions (green) and River dolphin (orange) alignment to rat (gray), represented by sticks.

Substitution of all 4 residues together or only their partial combination did not result in a tertiary structure identical to that of the deep diving cetaceans; illustrated here for Sperm whale (Figure 8). The sperm whale TMD conformation is more distorted compared to the rat possessing the same 4 substitutions. Rat TMDs possessing only 1–3 substitutions are even less distorted compared to the native rat. Similar predictions for the Zn^2+^ site and its 4 residues (Figure 9) did not conform to the Sperm whale spatial structure of the site, although they resembled the position observed for the E266 residue predicted for the River dolphin.

However, if we added Sperm whales’ two special NTD a.a. substitutions T156I and S224T we obtained a completely different picture. TMD conformation is more distorted compared to the rat with RMSD = 2.47 (Figure 8E). In Zn^2+^ site E266, has a rotation on the CG atom (reminiscent of Sperm whales’ rotation) and on the CB atom (96.06° reminiscent of River dolphins’ rotation) (Figure 9C). In addition, D282 has a rotation on CB atom of 79.81° and H128 does not align with rats’. (3D representation of the Zinc site alignment of the Sperm whale, River dolphin, and rat including six mutations is found in Supporting Information of this work, “Zinc site.pse”).

## Discussion

The purpose of this research was to disclose whether the breath-hold deep diving mammals exhibit NMDAR structural modifications related to HP environment. Examination of the GluN1 and GluN2B subunits sequences of the marine mammals revealed that they present no major change. Their tertiary structure is preserved and they align almost perfectly to those of the terrestrial mammals. In contrast, our research revealed that 3D of TMD of the GluN2A subunit, which is one of the abundant subunits in adult human CNS and plays a crucial role in LTP, of the marine mammals differs from that of the terrestrial mammals. There are four conserved a.a. substitutions (that appear in all members of the cetacean family) in the GluN2A subunit: V67L (in NTD), E308D (in NTD), V440I (in ABD), and I816V (in TMD, M4 α-helix) (Figure 4). Although E308D appears also in goat and rabbit, and I816V appears in camel, rabbit and manatee, we hypothesize that the presence of all four substitutions is the important factor, rather than a single substitution found in various terrestrial mammals. This was also shown by substituting those residues in rat sequence. Substitution of only one a.a. showed a very small change indicated by small RMSD (Figure 8), while a substitution of 2 a.a. in NTD and 4 conserved cetacean a.a. cause a much more significant change. We can conclude that the combination of four (two specific and two more general) substitutions, which appear in all cetacean mammals probably influence their GluN2A tertiary structure, but some additional amino acids substitutions, unique to each cetacean mammal, are necessary for determining the final structure of that subunit.

Alignment of GluN2A subunits of the diving mammals to those of rat showed that the NTD and ABD tertiary structures align very well and do not show major changes. On the other hand, TMD exhibited very different tertiary structure in some marine mammals (Figure 6). The biggest difference was observed in Sperm whale and Weddell seal, which are deep divers of their families. The most significant differences were observed in M1 and M4 α-helixes conformation. Recent studies have indicated that NMDAR function is strongly modulated by lipids interaction (Casado and Ascher, 1998; Korinek et al., 2015) and the M4 peripheral TMD α-helix segment, which interacts with the pore domain of the neighboring subunits, is critical for NMDAR activation and desensitization (Amin et al., 2017). Substitutions in its sequence may strongly affect some of the receptor biophysical properties. GluN2A subunit with different biophysical properties may affect the whole CNS (see section “Introduction”) and the way it functions and responds.

Although generally, marine mammals’ NTD aligns with a high score to that of the rat (average RMSD of 0.323) and it looks like there are no differences in their structure, close examination of the Zn^2+^ binding site prediction shows a rotation of the E266 in the Sperm whale (Figure 7). This special conformation may interfere with Zn^2+^ binding and therefore indicates the lack of Zn^2+^ voltage-independent inhibition of NMDAR in sperm whales. If so, it could serve as an explanation for whale capability of performing extremely deep dives of almost 2 km. It has been shown by our laboratory that the receptors containing the GluN2A subunit exhibit the biggest current increase at HP (Mor et al., 2012; Bliznyuk et al., 2016), whereas other subtypes showed slight inhibition or no change of the currents. In other words, with the lack of Zn^2+^ voltage-independent inhibition at the surface they will not experience removal of that inhibition at HP, and consequently will face much less increase of the current, resulting in less hyperexcitability of the CNS. Unfortunately sequence of the same region in Weddell seal is missing. We do not know if this is an actual biological deletion of a large portion or a technical fault of the sequence methods. It would be extremely valuable to compare the sequences of the Elephant seals (presently unavailable) who are the record divers among pinnipeds (>1500 m!), to see whether they also demonstrate any deletions in that Zn^2+^ site. If so, it may suggest that they also lack Zn^2+^ voltage-independent inhibition, yet attained by adopting a different “strategy.” This is even more striking since all the pinnipeds presently studied (Hawaiian monk seal, Weddell seal, and Florida manatee) do not exhibit the three or four substitutions in GluN2A that were observed in cetaceans.

One of the possible explanations of the unique conformation of the Zn^2+^ site in Sperm whale are the two special NTD a.a. substitutions T156I and S224T that do not appear in other marine or terrestrial mammals. Adding these substitutions, in addition to the four cetaceans’ substitutions, to the rat model dramatically changed its Zn^2+^ site structure (Figure 9). It is worth noticing that mutations of even a single residue in the NTD region in humans, for example P79R or R370W, results in altered Zn^2+^ sensitivity (Serraz et al., 2016). Thus, cetaceans 2 unique conserved substitutions in the NTD (V67L and E308D) may also alter zinc sensitivity, although diving mammals preserve the NTD sequence and most of them do not show special conformation of the Zn^2+^ site. The possible residual or lack of Zn^2+^ inhibition could be helpful in HP tolerance.

Several hypotheses have been suggested in order to explain diving mammals’ capabilities in coping with pressure and hypoxia. Higher percentage of protective glia and thus a lower percentage of neurons in deep diving mammals may explain their HP diving ability (Poth et al., 2005). Also, the different neuronal (or glia) membrane phospholipids composition, especially the cholesterol that has been shown as an important modulator of the NMDAR (Korinek et al., 2015), may play a special baroprotective role in their fitness to HP environment. In addition, diving mammal fatty tissues are saturated with N_2_ (Koopman and Westgate, 2012) that can play a role as a narcotic antagonist to the hyperexcitation effects. It was also suggested that high levels of corticosteroids could be correlated with deep diving capability of pinnipeds (phocids) as compared to shallow divers of pinnipeds (otariids) (Liggins et al., 1993).

It is conceivable that during evolution several physiological mechanisms were evolved in diving mammals to enable them breath hold diving in general. Yet, additional mechanisms, especially protective against HP environment effects, namely HPNS, should also be incorporated for deep diving foraging. We suggest that the special tertiary structure of the GluN2A, and possibly the lack of voltage- independent Zn^2+^ inhibition in the NMDAR is an important feature that may give marine mammals the advantage they need in order to perform such deep dives without CNS malfunction. In order to verify this hypothesis we need to directly measure the currents of their unique-sequence subunits.

## Author Contributions

AB designed the study, acquired the data, analyzed and interpreted the data, and drafted the manuscript. HG and YG interpreted the data and did the critical revision of the paper.

## Conflict of Interest Statement

The authors declare that the research was conducted in the absence of any commercial or financial relationships that could be construed as a potential conflict of interest.

## Supplementary Material

FIGURE S1 Sequence alignment of GluN1 subunit in different marine and terrestrial mammals.

FIGURE S2 Sequence alignment of GluN2B subunit in different marine and terrestrial mammals.

FIGURE S3 Sequence alignment of GluN2A subunit in different marine and terrestrial mammals.

## Abbreviations

A: Alanine
ABD: Agonist-binding domain
AMPAR: α-amino-3-hydroxy-5-methyl-4-isoxazolepropionic acid receptor
CNS: Central nervous system
CTD: C-terminal domain
D: Aspartic acid
E: Glutamic acid
G: Glycine
H: Histidine
HP: High pressure
HPNS: High Pressure Neurological Syndrome
I: Isoleucine
iGluRs: ionotropic glutamate receptors
L: Leucine
LTD: Long-term depression
LTP: Long-term potentiation
NMDAR: N-methyl-D-aspartate receptor
NTD: N-terminal domain
P: Proline
R: Arginine
RMSD: Root Mean Square Deviation
S: Serine
T: Threonine
TMD: Transmembrane domain
V: Valine
W: Tryptophan
