# Supplementary figures and images for: Marine Mammals’ NMDA Receptor Structure: Possible Adaptation to High Pressure Environment
Source: Front Physiol. 2018 Nov 22;9:1633. doi: 10.3389/fphys.2018.01633 (PMC6262034; doi:10.3389/fphys.2018.01633)

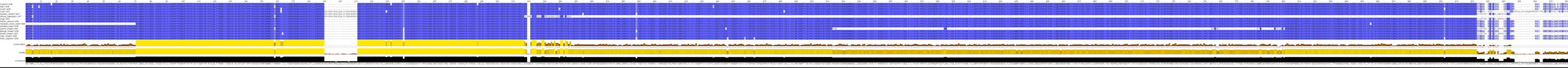

Supplement: FIGURE S1 — Sequence alignment of GluN1 subunit in different marine and terrestrial mammals. [file Image_1.PNG]

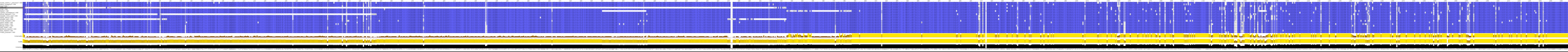

Supplement: FIGURE S3 — Sequence alignment of GluN2A subunit in different marine and terrestrial mammals. [file Image_3.PNG]
